# Self-injurious behaviour in patients with anorexia nervosa: a quantitative study

**DOI:** 10.1186/s40337-018-0214-2

**Published:** 2018-10-03

**Authors:** Linda Smithuis, Nienke Kool-Goudzwaard, Janneke M. de Man-van Ginkel, Harmieke van Os-Medendorp, Tamara Berends, Alexandra Dingemans, Laurence Claes, Annemarie A. van Elburg, Berno van Meijel

**Affiliations:** 1 Parnassia Psychiatric Institute, Rotterdam, The Netherlands; 2 Inholland University of Applied Sciences, Amsterdam, The Netherlands; 3 Intensive Treatment Centre, Fivoor, The Hague, The Netherlands; 4 Julius Center for Health Sciences and Primary Care, Department of Nursing Science, University Medical Center Utrecht, University Utrecht, Utrecht, The Netherlands; 5 Department of Dermatology, University Medical Center Utrecht, Utrecht, the Netherlands; 6 Department of Allergology, University Medical Center Utrecht, Utrecht, the Netherlands; 7 Altrecht Eating Disorders Rintveld, Zeist, The Netherlands; 8 Rivierduinen Eating disorders Ursula, Rivierduinen, Leiden, The Netherlands; 9 Institute of Psychology, Leiden University, Leiden, the Netherlands; 10 Faculty of Psychology and Educational Sciences, KU Leuven, Leuven, Belgium; 11 Faculty of Medicine and Health Sciences, University of Antwerp, Antwerp, Belgium; 12 Clinical Psychology, Altrecht Eating Disorders Rintveld, Zeist, The Netherlands; 13 Faculty of Social Sciences, Utrecht University, Utrecht, The Netherlands; 14 Mental Health Nursing, Inholland University of Applied Sciences, Amsterdam, The Netherlands; 15 Department of Psychiatry, Amsterdam Public Health Research Institute, VU University Medical Center, Amsterdam, The Netherlands; 16 Parnassia Psychiatric Institute, The Hague, The Netherlands; 17 GGZ-VS, Academy for Masters in Advanced Nursing Practice, Utrecht, The Netherlands

**Keywords:** Anorexia nervosa, Feeding and eating disorders, Self-harm, Self-injurious behaviour

## Abstract

**Background:**

Many patients with an eating disorder report difficulties in regulating their emotions and show a high prevalence of self-injurious behaviour. Several studies have stated that both eating disorder and self-injurious behaviour help emotion regulation, and are thus used as coping mechanisms for these patients. We aimed to determine the prevalence of self-injurious behaviour, its characteristics and its emotion-regulation function in patients with anorexia nervosa or an eating disorder not otherwise specified (*n* = 136).

**Methods:**

A cross-sectional design using a self-report questionnaire. Mann–Whitney *U*-tests were conducted to compare the background and clinical variables between patients with self-injurious behaviour and patients without this type of behaviour. Changes in emotional state before and after self-injurious behaviour were tested by Wilcoxon signed rank tests.

**Results:**

Our results showed a 41% prevalence of self-injurious behaviour in the previous month. Patients who performed self-injurious behaviour had a statistically significant longer treatment history for their eating disorder than those who did not. Whereas 55% of self-injuring patients had a secondary psychiatric diagnosis, only 21% of participants without self-injurious behaviour did. Regarding the impact of self-injurious behaviour, our results showed a significant increase in “feeling relieved” and a significant decrease in “feeling angry at myself”, “feeling anxious” and “feeling angry at others”. This indicates that self-injurious behaviour can be regarded as an emotion-regulation behaviour. Participants were usually aware of the causes of their self-injurious behaviour acts.

**Conclusions:**

Professionals should systematically assess the occurrence of self-injurious behaviour in eating disorder patients, pay special attention to patients with more severe and comorbid psychopathology, and those with a long treatment history. This assessment should be followed by a functional analysis of the self-injurious behaviour and by effective therapeutic interventions alongside the eating disorder treatment.

## Plain English summary

Difficulties in the regulation of emotions are common among people with an eating disorder. The use of self-injurious behaviour may occur as a coping strategy for overwhelming emotions. We investigated the prevalence and the emotion-regulation function of this behaviour in patients with anorexia nervosa or an eating disorder not otherwise specified. The prevalence of self-injurious behaviour in the previous month was 41%. We found that patients who performed self-injurious behaviour had been in treatment longer for their eating disorder and were more likely to have a secondary psychiatric diagnosis that may indicate more severe pathology than patients who do not self-injure. After an act of self-injurious behaviour, many experienced a reduction in negative feelings and an increased feeling of relief, and were able to articulate emotions which prompted the act of self-injury. Knowing the precursors of self-injury is helpful for behavioural change and to help find alternative strategies.

## Background

An eating disorder (ED) is a severe mental illness that seriously affect a person’s physical and mental health. The main categories in DSM-5 of EDs are Anorexia Nervosa (AN); Bulimia Nervosa (BN); and Binge Eating Disorder (BED). Another category, Other Specified Feeding or Eating Disorder (OSFED), applies to eating-disordered behaviours which do not meet the full criteria for any of the disorders in the feeding and eating disorders diagnostic class [1].

Emotion-regulation difficulties are reported in all types of EDs [2]. Emotion regulation refers to the methods of attending, evaluating and modifying emotional states [3]. Higher levels of emotion dysregulation are related to more serious eating disorder symptoms [4]. Several studies have theorized that the symptoms of AN, such as food restriction, excessive exercise and purging behaviour, represent attempts to regulate emotional states [5–7]. Emotion regulation seems to differ between subtypes of AN: AN patients with the purging subtype have reported greater difficulties with emotion regulation than patients of the restrictive [8]. Although those with BN have reported greater difficulties with emotion regulation than controls [2, 9–11], previous studies have found no significant differences between AN patients and BN patients regarding emotion dysregulation [2, 9–11].

To regulate intense and sometimes overwhelming emotions, ED patients may engage in self-injurious behaviour (SIB) as a coping strategy [12–14]. SIB has been defined as the deliberate damage of one’s body tissue without suicidal intent [15]. Various studies have shown that it is highly prevalent among patients with ED, with prevalence rates ranging from 25.4 to 55.2% in all types of EDs [16–18]. A life-time prevalence of 27.3% for any type of ED and 21.8% for AN was found in the review by Cucchi et al. [19]. Some authors state that the SIB and disturbed eating patterns in these patients both function as a coping strategy for dealing with burdensome emotional states. This may explain the high prevalence of SIB in ED [20, 21].

A study examining the differences between ED patients with and without SIB found that patients with SIB had a higher severity of disordered eating behaviour [22]. ED patients with SIB also had more difficulties with emotion regulation. The use of multiple methods of SIB was also found to be associated with more severe eating-disorder symptoms [22].

Due to the high prevalence of SIB in patients with an ED, Claes et al. [17] studied the emotion-regulation function, examining the changes in emotional state before and after SIB, and also the predictive value of emotional states for the frequency and chronicity of SIB [17]. For all methods of SIB, they found a consistent pattern of changes in emotional states, which was categorized in four groups based on two dimensions [23]: (1) valence, that is, the pleasantness of an emotion (positive vs negative); and (2) arousal, that is, the intensity of an emotion (high vs low). Whereas positive-low arousal affect (such as feeling relieved) increased significantly after SIB, negative-high-arousal affect (such as anxiety) decreased significantly. On the basis of this and other studies [24–26], it could be concluded that SIB is a functional behaviour whereby a patient regulates emotions in the short term.

To increase their understanding of SIB in patients with an ED, Claes and Muehlenkamp [27] developed a conceptual model of the interactive risk factors for both SIB and EDs. The model depicts risk factors on two dimensions: (1) distal factors, that is, individual risk factors and social risk factors; and (2) proximal factors, that is, emotional dysregulation, cognitive distortions, low body regard, dissociation and psychiatric disorders. When combined with stressful events, interaction between the distal and proximal risk factors can increase emotional distress. To regulate this distress, SIB and/or eating-disordered symptoms may take place, which can in turn increase the occurrence of the proximal risk factors. This interplay contributes to a complex reciprocal pattern involving emotional dysregulation, SIB and eating disordered behaviour [27].

Although earlier studies [14, 16–18, 20, 22, 27] have shown that SIB is common in patients with an ED, we are unaware of any studies on the prevalence and nature of SIB among ED patients in the Netherlands. On the basis that the conceptual model of Claes and Muehlenkamp [27] might demonstrate the applicability of these earlier studies, we aimed to improve our understanding of SIB in patients with EDs by investigating SIB rates in ED patients and consider these alongside those found in a number of other countries [16–18, 20, 27]. Further, we aimed to explore emotional functions of that SIB Collectively, these goals may improve awareness of and treatment of SIB in patients with an ED and help healthcare professionals and patients gain insight into the characteristics and emotion-regulation function of SIB.

### Aim

In this Dutch study, we therefore aimed (1) to identify the prevalence and characteristics of SIB in patients with an ED, (2) to investigate the differences in background variables among ED patients with and without SIB, and (3) to investigate the emotion-regulation function of SIB for patients with an ED.

## Methods

### Design

This study was based on a cross-sectional design, and used a self-report questionnaire to measure the prevalence and characteristics of SIB among patients with an ED [28].

The study was approved by the research committee at one of the treatment centres (protocol number 1140), in accordance both with the declaration of Helsinki [29] and with the Dutch legislation regarding medical research in health care. Based on this approval, the board of directors of the second participating organization gave permission for the inclusion of patients from their organization.

### Study setting and participants

Data were obtained in the Netherlands at two specialized treatment centres for patients with an ED. Convenience sampling was used to approach as many participants as possible in the treatment centres during the period of the study, from May 2011 till September 2011 [30]. In face-to-face meetings, inpatients and outpatients who participated in group treatment were informed about the purpose and procedures of the study and were invited to participate. Outpatients who received individual treatment received information about the study by letter and were also invited to participate.

We included patients aged 16 years and older who had a primary diagnosis of an ED according to DSM IV: i.e. anorexia nervosa, bulimia nervosa or an eating disorder not otherwise specified (EDNOS). Patients were excluded if the psychiatrist determined on the basis of clinical assessment that participation might be too burdensome. In total, 372 patients were approached for participation, 158 of whom returned the questionnaire, representing a response rate of 42.5%.

The index diagnoses (according to the DSM-IV criteria) were determined by the multidisciplinary team of clinicians (psychologists and psychiatrists), using questions from two standardized semi-structured interviews: the Eating Disorder Examination [31] and the Longitudinal Interval Follow-up Evaluation [32]. Diagnoses were discussed with each patient at the end of the intake procedure and described in the treatment plan, which was made available to the patient. Based on this information, patients reported on their current psychiatric diagnoses.

The study included only participants with AN or EDNOS, in accordance with the DSM-IV that was used at the time of inclusion. We excluded BN patients (*n* = 22). The number of BN patients with SIB was very limited in our sample (*n* = 6) and constituted a different subgroup than patients with AN and EDNOS. By limiting ourselves to AN and EDNOS, the sample was more homogeneous: in the studies of Serrano-Troncoso et al. [33]; Vo et al. [34] and Keel et al. [35], most cases of EDNOS were eventually diagnosed as AN-type according to the DSM-5, which has been used in clinical practice since 2017.

All participants voluntarily agreed to participate in the study and gave their written informed consent. Written informed consent for participants aged younger than 18 years was obtained by their parents.

### Measures and procedures

The participants completed the questionnaire at home and returned it by post. Through self-report, the following background and clinical variables were obtained: age, gender, height, weight, living situation, inpatient/outpatient status, other psychiatric diagnoses, type of eating disorder, duration of eating disorder, and treatment duration over the course of the ED specifically.

We used the validated Dutch version of Self-Injury Questionnaire-Treatment Related (SIQ-TR) [36], which was originally designed to assess five different subtypes of SIB: “How long ago did you (1) scratch, (2) bruise, (3) cut, (4) burn and (5) bite yourself?” to be answered on the following scale “a week, a month, a year, over a year, or never”. The internal consistency of these five types of SIB in this questionnaire was α = 0.62, indicating that the different types were moderately related. The addendum to the questionnaire gave participants an opportunity to describe any additional type of SIB that could not be categorized under the main five subtypes. For each of the five subtypes of SIB separately it was asked how long since the behaviour had been performed: a week, a month, a year, over a year, or never. If the SIB had taken place in the previous month, the following SIB characteristics were obtained: whether the act of SIB had been planned beforehand; whether the patient was aware what had caused SIB; whether he or she had taken care of the wounds; and whether he or she had hidden the SIB from others. Each follow-up question could be answered on a four-point scale: 1 = never, 2 = sometimes, 3 = often, 4 = always.

The reasons for SIB were assessed on the basis of 11 statements that were scored on a scale ranging from 1 (not at all) to 5 (very much). To investigate the emotion-regulation function of SIB, participants were asked to rate the severity of nine emotional states before and after SIB on a scale ranging from 1 (not at all) to 5 (very much). Due to the cross-sectional nature of the questionnaire, the emotional states before and after performing SIB was scored retrospectively on the basis of the participant’s memory.

### Data analysis

To determine the prevalence rates of SIB and the SIB characteristics, frequencies were calculated for the 136 patients (42.5%) who completed the questionnaires.

Differences between the two subgroups (SIB in the last month and never performed SIB) with respect to background and clinical variables were determined on the basis of Mann–Whitney *U*-tests. For categorical data, the chi-square test statistic was used. Changes in emotional state before and after SIB were tested by Wilcoxon signed rank tests.

The statistical significance level for all tests was set at *p* < 0.05. Effect sizes for Mann–Whitney *U*-tests and Wilcoxon signed rank tests were expressed in *r*, with *r* = .10 small effect; *r* = .30 medium effect; and *r* = .50 large effect [37, 38]. Statistical analyses were conducted using SPSS version 24 [IBM, Armonk, NY, USA].

## Results

### Sample characteristics

Table 1 summarise the sample characteristics. In total, 136 participants with AN or EDNOS were included. Most participants were female (*n* = 131, 96%) with a mean age of 25 years ((SD) 8.55; range 16–58 years). Ninety-eight participants (72%) had AN. EDNOS was diagnosed in 38 participants (28%). Most participants (*n* = 114, 84%) were outpatients. Fifty-six reported a comorbid psychiatric diagnosis (41%), the three most common being post-traumatic stress disorder (*n* = 15, 11%), personality disorder not otherwise specified (*n* = 13, 10%), and depressive disorder (*n* = 12, 9%).

**Table 1:** Sample characteristics

Variable	Total (*n* = 136)	AN (*n* = 98)	EDNOS (*n* = 38)
Age, years *M [SD]*	25.44 [8.56]	24.12 [7.13]	28.84 [10.83]
Gender female, *n [%]*	131 [96.3]	96 [98.0]	35 [92.1]
Body mass index *M [SD]*	19.48 [5.53]	18.02^a^ [3.45]	20.52^b^ [3.14]
ED duration, years *M [SD]*	8.4 [7.8]	7.77 [6.70]	10.17 [10.03]
ED treatment duration, years *M [SD]*	3.2 [3.4]	3.59 [3.65]	2.27 [2.38]
Comorbid psychiatric diagnosis *n [%]*			
No comorbid diagnosis	80 [58.8]	59 [60.2]	21 [55.3]
Comorbid diagnosis total	56 [41.2]	39 [39.8]	17 [44.7]
PTSD	15 [11.0]	13 [13.3]	2 [5.3]
Personality disorder NOS	13 [9.6]	9 [9.2]	4 [10.5]
Depressive disorder	12 [8.8]	8 [8.2]	4 [10.5]
Anxiety disorder	6 [4.4]	3 [3.1]	3 [7.9]
Obsessive compulsive disorder	3 [2.2]	2 [2.0]	1 [2.6]
Borderline personality disorder	3 [2.2]	2 [2.0]	1 [2.6]
Asperger syndrome	2 [1.5]	1 [1.0]	1 [2.6]
Alcohol addiction	1 [0.7]	–	1 [2.6]
Panic disorder	1 [0.7]	1 [1.0]	–
Treatment program *n [%]*			
Inpatient	22 [16.2]	19 [19.4]	3 [7.9]
Outpatient	114 [83.8]	79 [80.6]	35 [92.1]
Living situation *n [%]*			
Alone	43 [31.6]	28 [28.6]	15 [39.5]
With partner or friend[s]	32 [23.5]	22 [22.4]	10 [26.3]
With parents	54 [39.7]	42 [42.9]	12 [31.6]
Sheltered living	2 [1.5]	1 [1.0]	1 [2.6]
Other	5 [3.7]	5 [5.1]	–
Performing SIB *n* [%; 95%CI]			
Lifetime	83 [61; 53.1–69.4]	61 [62.9; 53.0–72.0]	22 [57.9; 42.1–72.5]
Last year	68 [50.0; 47.7–58.3]	48 [49.0; 39.2–58.8]	20 [52.6; 37.1–67.8]
Last month	56 [41.18; 50.4–66.8]	38 [38.8; 29.6–48.6]	18 [47.4; 32.2–62.9]

*AN* Anorexia Nervosa restrictive subtype and anorexia nervosa binge eating/purging subtype, *EDNOS* eating disorder not otherwise specified, *ED* eating disorder, *BMI* body mass index, *PTSD* post-traumatic stress disorder, *Personality disorder NOS* not otherwise specified

^a^Three missing values for BMI and 1 outlier for BMI < 42.73>

^b^One missing value for BMI and 5 outliers for BMI range < 32.41–44.29>

### Prevalence of self-injurious behaviour

The lifetime prevalence of SIB in our sample was 61% (*n* = 83; 95% CI: 53.1–69.4). During the previous year, 50% (*n* = 68) had performed at least one type of SIB (95% CI: 47.7–58.3). In the month preceding the study, the prevalence of SIB had been 41% (*n* = 56; 95% CI: 50.4–66.8).

A total of 108 acts of SIB within the past month were reported by 56 participants and consisted of seven different types: cutting (59%, performed by *n* = 33); scratching (46%; *n* = 26); bruising (34%; *n* = 19); biting (20%; *n* = 11); burning (14%; *n* = 8); hair-pulling (11%; *n* = 6); and hitting (9%; *n* = 5). One type of SIB was reported by 37% (*n* = 21) of the participants, two types by 39% (*n* = 22); three types by 18% (*n* = 10); four types by 4% (*n* = 2); and five types by 2% (*n* = 1). Over half the participants (*n* = 35; 62%) thus reported having performed two or more types of SIB in the previous month.

### Background and clinical variables

Mann–Whitney *U*-tests were conducted to compare the background and clinical variables of participants who had performed SIB in the previous month (*n* = 56) with those who never performed SIB (*n* = 53). The results indicated that participants who had performed SIB in the previous month had a significantly longer treatment history for their ED (*Mdn* = 3.00, *IQR* 1.0–5.0) than those who never performed SIB (*Mdn* = 1.00, *IQR* 0.5–3.0), *U* = 888.50, *z* = − 3.63, *p* < .001.

The chi-square tests between these groups on having a comorbid psychiatric diagnosis was found to be statistically significant (χ^2^(1) = 13.77, *p* < .001): over half of the participants (55%) who had performed SIB in the previous month had a comorbid diagnosis, compared to 21% of patients who never performed SIB.

There were no statistically significant differences between the two groups regarding age, gender, body mass index, primary ED diagnosis, duration of the eating disorder in years, or inpatient/outpatient and living situation. See Tables 2 and 3 for more detailed information.

**Table 2:** Comparison between patients with SIB in last month and never performed SIB

Background variable	Group	*n*	*Median*	*IQR*	Test-statistic *U*^a^	z	*p*-value
Age	SIB	56	22.00	19.00–26.00	1192.00	−1.78	.08
	Non-SIB	53	24.00	20.50–28.00			
BMI	SIB	56	19.10	17.66–20.11	1329.50	−0.63	.53
	Non-SIB	53	18.63	15.77–20.19			
ED in years	SIB	56	6.00	3.00–10.00	1298.00	−1.13	.26
	Non-SIB	53	4.00	3.00–8.25			
ED treatment in years	SIB	56	3.00	1.00–5.00	888.50	−3.63	**<.001**
	Non-SIB	53	1.00	0.50–3.00			

*SIB* Self-injurious behaviour, *ED* eating disorder, *BMI* body mass index, *IQR* inter quartile range; *p*-value in bold significant result at level *p* <.05

^a^Mann–Whitney *U*-test

**Table 3:** Comparison between patients with SIB in last month and never performed SIB

Background variable	SIB (*n* = 56)	No SIB (*n* = 53)	Test statistic^a^ χ^2^	*p*-value
Female gender %	96.4	94.3	0.27	.60
ED diagnosis AN %	67.9	69.8	0.05	.83
Patients in outpatient programme %	80.4	86.8	0.82	.37
Secondary diagnosis yes%	55.4	20.8	13.77	**<.001**
Patients living alone %	28.6	34.0	0.37	.54

*SIB* Self-injurious behaviour, *ED* eating disorder; *p*-value in bold significant result at level *p* <.05

^a^Pearson chi square

### Characteristics of self-injurious behaviour in patients with an eating disorder

Table 4 presents more detailed information on the characteristics of the SIB behaviour.

**Table 4:** Characteristics of Self-injurious Behaviour

Attitude	All acts (*n* = 107)	Individual participant (*n* = 56)
Behaviour was planned beforehand		
Never	57.9%	51.8%
Sometimes	36.4%	39.3%
Often	5.6%	8.9%
Always	0	0
Patient aware of how it had come about		
Never	15.0%	12.5%
Sometimes	48.6%	46.4%
Often	32.7%	39.3%
Always	3.7%	1.8%
Patient took care of the wounds		
Never	34.6%	21.4%
Sometimes	14.0%	19.6%
Often	12.1%	16.1%
Always	39.3%	42.9%
Patient hid the act of SIB from others		
Never	2.8%	1.8%
Sometimes	14.0%	17.9%
Often	27.1%	30.4%
Always	56.1%	50.0%

*SIB* Self-injurious behaviour

Of the participants who had performed SIB in the past month, 58% of the total number of registered SIB acts had not been planned beforehand. Participants were sometimes (46%) or often (39%) aware of what had caused the SIB act. Over half of the SIB acts had remained hidden from others (56%).

### Reasons for self-injurious behaviour

Table 5 presents more detailed information about the motives for SIB. The most important reasons for SIB were “to avoid negative feelings” (*M* = 3.82, *SD* = 1.30) and “to punish myself” (*M* = 3.89, *SD* = .1.42). The reason “to get attention from others” was hardly mentioned (*M* = 1.23, *SD* = .0.50).

**Table 5:** Reasons for self-injurious behaviour

Reasons for SIB M [SD]	All acts (*n* = 107	Individual participant (*n* = 56)
To feel some pleasure	1.64 [1.12]	1.52 [0.99]
To avoid or suppress negative feelings	4.05 [1.19]	3.82 [1.30]
To avoid or suppress painful images or memories	2.99 [1.71]	2.84 [1.76]
To get into a twilight or numb state	2.36 [1.46]	2.36 [1.51]
To get attention from others	1.18 [0.43]	1.23 [0.50]
To escape from a twilight or numb state	1.78 [1.27]	1.66 [1.21]
To punish myself	4.15 [1.28]	3.89 [1.42]
To make myself unattractive	1.67 [1.17]	1.64 [1.17]
To avoid or suppress suicidal thoughts	2.56 [1.54]	2.48 [1.54]
To show myself how strong I am	2.32 [1.46]	2.16 [1.40]
To show others how strong I am	1.41 [0.90]	1.34 [0.86]

The range for the assessment of each reason is 1 = not at all; 2 = a bit; 3 = moderately; 4 = much; 5 = very much

### The function of self-injurious behaviour in the emotional state

Table 6 describes the emotional states before and after SIB and the changes in emotional states.

**Table 6:** Changes in emotional state after self-injurious behaviour

Emotional state^a^	All SIB acts^b^ (*n* = 107)					Individual participant (*n* = 56)				
	Before	After	*z*-value	*p*-value^b^	*r*	Before	After	*z*-value	*p*-value^b^	*r*
Feeling happy	1.00 [1.00–1.00]	1.00 [1.00–1.00]	−4.27^c^	<.001	−.29	1.00 [1.00–1.00]	1.00 [1.00–1.00]	−2.49^c^	.013	−.24
Feeling relieved	1.00 [1.00–1.00]	3.00 [2.00–4.00]	−7.89^c^	**<.001**	−.54	1.00 [1.00–1.00]	2.50 [2.00–4.00]	−5.83^c^	**<.001**	−.55
Feeling nervous	2.00 [1.00–3.00]	2.00 [1.00–3.00]	−1.05^d^	.29	−.07	2.50 [1.00–3.00]	2.00 [1.00–3.00]	−1.28^d^	.20	−.12
Feeling bored	1.00 [1.00–1.00]	1.00 [1.00–1.00]	−2.92^d^	.003	−.20	1.00 [1.00–2.00]	1.00 [1.00–1.00]	−1.80^d^	.07	−.17
Angry at myself	5.00 [4.00–5.00]	4.00 [3.00–5.00]	−5.70^d^	**<.001**	−.39	5.00 [4.00–5.00]	3.50 [2.00–5.00]	−3.53^d^	**<.001**	−.33
Angry at another	2.00 [1.00–3.00]	1.00[1.00–2.00]	−4.81^d^	**<.001**	−.33	2.00 [1.00–3.75]	1.00 [1.00–2.00]	− 3.94^d^	**<.001**	−.37
Feeling anxious	4.00 [2.00–5.00]	3.00 [2.00–4.00]	−3.84^d^	**<.001**	−.26	4.00 [2.00–4.00]	3.00 [2.00–4.00]	−3.60^d^	**<.001**	−.34
Feeling sad	4.00 [3.00–5.00]	4.00 [3.00–5.00]	−4.25^d^	<.001	−.29	4.00 [3.00–5.00]	3.50 [2.00–4.75]	−2.96^d^	**.003**	−.28
Feeling guilty	4.00 [2.00–5.00]	4.00 [3.00–5.00]	−1.05^c^	.30	−.07	3.00 [2.00–5.00]	4.00 [2.25–5.00]	− 1.09^d^	.28	−.10

The range for the assessment of each emotional state is 1 = not at all; 2 = a bit; 3 = moderately; 4 = much; 5 = very much; *p*-value in bold is statistically significant and clinically relevant change

*SIB* Self-injurious behaviour

^a^Variables are denoted as median [inter quartile range]

^b^Differences in emotional state before and after SIB were tested with the Wilcoxon signed rank test

^c^based on negative ranks

^d^based on positive ranks

To investigate the function of SIB in emotional changes before and after an act of SIB, we analysed all acts together (*n* = 107). The results showed statistically significant reduction in the intensity two emotions, “feeling angry at myself” (*z* = − 5.70, *p* < .001, *r* = −.39); and “feeling angry at another” (*z* = − 4.81, *p* < .001, *r* = −.33); both of which had medium effect sizes. The intensity of “feeling anxious” also decreased significantly with a small effect size (*z* = − 3.84, *p* < .001, *r* = −.26). However, the intensity of the emotion “feeling relieved” increased significantly (*z* = − 7.89, *p* < .001) with a large effect size (*r* = −.54).

We also investigated the function of SIB with respect to emotional changes before and after SIB at the level of individual patients (*n* = 56). At this individual level, the intensity of the emotional state “feeling sad” reduced significantly (*z* = − 2.96, *p* < .003), with a small effect size of *r* = −.28. In line with the analyses regarding all SIB acts together, we also a found significant decrease in the intensity of “feeling angry at myself” (*z* = − 3.53, *p* < .001, *r* = −. 33); “feeling angry at another”, (*z* = − 3.94, *p* < .001, *r* = −.37); “feeling anxious” (*z* = − 3.60, *p* < .001, *r* = − 0.34); and a significant increase in the intensity of “feeling relieved” (*z* = − 5.83, *p* < .001, *r* = − 0.55) in our analysis on the individual patient level.

## Discussion

The present study describes the prevalence of SIB and the SIB characteristics in a sample of 136 Dutch patients with AN or EDNOS who received treatment in a specialized treatment setting for eating disorders.

The SIB prevalence rates over the past month and past year for Dutch patients with AN or EDNOS are in line with those shown in previous studies of ED patients [16–18, 22] but the reported life-time prevalence was higher compared to other studies. In the review of Cucchi et al. [19] a much lower overall life-time prevalence of 21.8% for AN was found. However, when differentiating between subgroups, it was found in this review that participants recruited from specialist ED or residential treatment settings were 2.6 times more likely to report a history of SIB than participants from general practices or community settings (OR 2.58, 95% CI 1.29–5.19, *p* < .01) [19]. Our sample contains only participants from specialized treatment settings, so this may explain the high life-time prevalence of SIB.

Cutting and scratching were the most common types of SIB; this, too, was consistent with previous studies [16, 17]. A large proportion of patients – 62% – performed more than one type of SIB, which was substantially higher than the proportion of 40% found in the study of Claes and Vandereycken [36].

Our results also showed that 55% of the participants who performed SIB had a comorbid psychiatric diagnosis. This high comorbidity was also found in other studies [17, 27]. Furthermore, Vieira et al. [22], who found in their study that SIB patients with EDs was associated with higher severity of ED symptoms. The participants in our own study reported a longer ED-treatment duration than those who never performed SIB. If these findings are taken together, it can be hypothesized that the pathology of ED patients with SIB is more severe, and that this may add to a longer treatment duration.

In over half of the manifestations of SIB, the act of SIB was not planned beforehand, although most participants mentioned that they were usually aware of how the SIB act had come about. The function of SIB they frequently reported was to avoid negative feelings and to punish oneself. This implies that SIB was usually an unplanned act, but eventually an acting out behaviour by increased negative emotions. This is in line with findings of other studies [14, 17, 23, 36].

According to both the literature [17, 20, 27] and our results, SIB plays a strong role in regulating negative emotions; because feeling relieved increases after SIB, SIB is negatively reinforced, which may explain why the SIB has a recurrent and persistent pattern for many [14, 27]. Considering our findings regarding the emotion-regulating functions of SIB, in combination with the reported functions of avoidance of negative feelings and the urge to self-punishment, it indicates that SIB can be regarded as a strong functional behaviour by patients.

### Strengths and limitations

To the best of our knowledge, this is the first study to be conducted in the Netherlands that aims to identify the prevalence of SIB in Dutch ED patients in treatment. To create a homogeneous group and thus generalizability, we included only those with AN and EDNOS according to DSM IV. We can draw no conclusions regarding patients with other EDs such as BN and BED. Also, given the specific nature of our sample, the findings cannot be generalized beyond specialized treatment centers for ED.

Some limitations should be considered. First, the non-response is considerable and the specific reasons for the non-response (57.5%) are unknown. In the scientific literature no consensus exists on acceptable (non-) response rates [39], though a response rate of more than 40% is considered acceptable for drawing valid conclusions for the population represented by the responders of our sample. It should be noted that the prevalence rates found in our study are in line with previous studies. So, non-response bias may have occurred, but the specific direction of this bias cannot not be determined in this present study*.*

Second, due to the cross-sectional design of the study and the retrospective nature of the questionnaire, recall bias may have significantly affected the participants’ evaluation of the emotions before and after SIB, and also of the reasons they gave for a SIB act. To reduce this, however, we asked participants to evaluate these items only if the SIB had taken place in the previous month.

Third, to ensure sufficient statistical power we analysed the total number of SIB acts. For participants who performed more than one type of SIB occur several times in these data, the emotion-regulation function for all the SIB acts together may be overrated: several SIB acts may have had the same function for the same participant.

### Relevance for clinical practice

Healthcare professionals treating patients with an ED should systematically assess the occurrence of SIB, paying special attention to those with more severe psychopathology, with respect to both the ED and co-morbidities. They should also explore the strong emotion-regulation function of SIB. Due to possible reciprocating interplay between SIB and the ED, therapeutic interventions should focus not only on emotion regulation, but also should identify any other coping mechanism beside SIB and eating-disordered behaviour. Preventive interventions focusing on increased coping and emotion regulation skills might also be applied.

For treatment of SIB to be effective, it is important that patients gain insight into the triggers that can lead to an act of SIB. Knowing the causes of SIB is a helpful adjunct for promoting behavioural change [40] including for therapeutic interventions based on cognitive behavioural therapy (CBT) which specifically aim to help patients gain a better understanding of these triggering factors and their consequences [40, 41].

In a phenomenological study, Verschueren et al. [14] described the process of how different triggering factors can lead to an act of SIB in patients with ED. Two important phenomena in this process were (1) being overwhelmed by emotions and [(2) the need to punish oneself. The fact that participants in our study did not refer to SIB as a technique for seeking attention, is consistent with previous studies [17, 23, 36]. Several studies among healthcare professionals have found that some healthcare professionals view attention-seeking is a strong component of patients’ performance of acts of SIB [42, 43]. Where this arises, this may stand in the way of providing optimal care and contribute to patients being less willing to talk openly about SIB and the emotions associated with it [14, 42, 43].

Due to its high prevalence in patients with an ED, SIB needs to be a topic of conversation between healthcare professional and patient [14, 42, 43]. Patients should thus be invited to communicate openly about their emotions and behaviour without shame or fear of rejection. Together, patient and healthcare professional can explore other coping strategies for dealing effectively with confusing thoughts and overwhelming emotions [14, 42, 43].

Future research could assess the prevalence of SIB (and its associated factors) in patients with eating disorders in treatment settings across different treatment intensities. In addition, future research should focus on the emotion-regulation capabilities of two groups of ED patients: those with SIB and those without. Finally, due to the complicated comorbidity of SIB in patients with an ED, research is needed to establish evidence on the efficacy of SIB interventions within this population.

## Conclusions

Our findings show a high prevalence of SIB in patients with AN or EDNOS. Patients who perform SIB have a longer ED-treatment history; half of them also have a comorbid psychiatric diagnosis that may indicate severe pathology. After an act of SIB, many patients experienced a reduction in several negative emotions such as anger towards themselves, which were then followed by greater relief. This indicates that SIB can be regarded as a functional emotion-regulation behaviour for patients.

## Abbreviations

AN: Anorexia nervosa
BED: Binge Eating Disorder
BN: Bulimia nervosa
CBT: Cognitive behavioural therapy
DBT: Dialectical behaviour therapy
ED: Eating disorder
EDNOS: Eating disorder not otherwise specified
OSFED: Other specified feeding and eating disorder
SIB: Self-injurious behaviour
SIQ-TR: Self injury questionnaire – treatment related
